# Fundamental Considerations and Analysis of the Energy Distribution in Laser Turning with Ultrashort Laser Pulses

**DOI:** 10.3390/mi14101838

**Published:** 2023-09-27

**Authors:** Julian Zettl, Cemal Esen, Ralf Hellmann

**Affiliations:** 1 Applied Laser and Photonics Group, University of Applied Sciences Aschaffenburg, 63743 Aschaffenburg, Germany; 2 Applied Laser Technologies, Ruhr University Bochum, 44780 Bochum, Germany

**Keywords:** ultrashort pulse laser, laser turning, tangential processing, trepanning optic

## Abstract

This article discusses the process of the laser turning of rotational symmetric, cylindrical components using ultrashort laser pulses with respect to the geometrical conditions and the resulting energy distribution during the laser turning process. As a result, process predictions and potential process optimizations are feasible. Particular attention is drawn to the laser spot formation on the cylindrical surface of the work piece in conjunction with the positioning of the laser beam relative to the rotation axis of the specimen. Based on fundamental calculations and experimental results, an optimum processing strategy is discussed, whereat the use of a trepanning optic in the laser turning process and the forming of a particular surface structure is additionally being issued.

## 1. Introduction

Laser ablation using ultrashort laser pulses offers the possibility of machining a large variety of materials, some of which are hard to machine using conventional approaches because of their material properties, for instance, the brittleness of fused silica [1,2,3], sapphire [4,5,6,7] or tungsten alloys [8,9,10]. This capability enables, for example, the manufacturing of high-precision microtools via the tangential processing of cylindrical work pieces [11,12]. As the process of laser turning with ultrashort laser pulses has recently gained more attention [13,14,15,16,17,18,19,20], a closer look at the geometrical situation and ablation behavior throughout the process is of high interest; yet, it has not been studied before.

Considering laser micromachining using ultrashort laser pulses, the positioning of the focal plane with respect to the material’s surface is of the upmost importance, since defocused processing may result in irregular results and excessive heat input into the material. There are various techniques and approaches to sustain an optimal distance between the focusing optic and material in order to keep the smallest focal diameter possible like, for example, machine vision-based focus detection and dynamic focus shift [21,22,23]. Applying tangential laser irradiation of the work piece in the laser turning process favors increased processing control because of the self-limitation of the ablation depth and allows for a high repeat accuracy, even for inhomogeneous materials [19]. Because of the geometrical conditions within the laser turning process, namely, the cylindrical shape of the work piece, the smallest possible focal spot size cannot be reached when processing tangentially. The impact on the spot’s shape on the material when applying a nonzero lead angle on a flat surface was presented by Wang et al. [24]. It is stated that the laser-irradiated area increases according to the lead angle by a factor of $1/\cos\left(\theta \right)$, where θ is the applied angle. It was found that different irradiating angles in the process result in a variation of the material’s removal rate, as well as in the surface roughness. A similar study by Moreno et al. [25], in which the effect of the oblique incident angles of the laser beam on the ablation rate of the painted flat surfaces was evaluated, drew the same conclusion. These studies, however, are merely representative for processing flat surfaces and only provide a rough prospect of the laser machining of a nonflat surface. In addition, the altered pulse-to-pulse overlap caused by the distorted laser projection on the surface and the associated effects were analyzed superficially. On cylindrical surfaces, the curvature of the specimen needs to be taken into account when calculating the resulting area and, therefore, the energy density on the surface. The resulting irradiated area on a cylindrical surface causes a notable change in the pulse-to-pulse overlap that severely affects the processing result.

Furthermore, combining the laser turning process with a trepanning optic not only offers an ablation rate benefit, as experimentally shown in [16,17], it also strongly influences the surface formation upon ablation, as demonstrated in this contribution. Although trepanning optics are mainly used in laser drilling operations [26,27,28,29,30], the advantages of fast optical beam deflection can be used in the laser turning process as well; yet, it has not been reported so far. This approach, however, results in a complex processing pattern where many factors play an important role. Based on the initial diameter of the material, the rotation speed, the pulse repetition rate, the trepanning diameter, the trepanning speed, the linear feed rate and the focused beam propagation properties, the processing result differs for each variation of one of these parameters. An important basic consideration when choosing a set of processing parameters is to ensure that there are no areas on the material’s surface that experience an excessive amount of laser irradiation or fluence, since this may result in severe irregularities. Because of the number of applicable parameters, a predetermination of the laser tool path is rather intricate. Moreover, because of the curvature of the work piece, a variety of laser spot areas and, therefore, fluences occur when applying a trepanning optic in the laser turning process.

In summary, while laser turning using ultrashort pulsed lasers can clearly be considered an attractive alternative for processing rotationally symmetric microcomponents, a thorough understanding of the energy deposition, particularly when applying additional trepanning optics, and the resulting ablation behavior is necessary. Against this background, in this contribution the impact of selected processing parameters on the material removal rate, as well as on the surface formation, during laser turning using ultrashort pulsed lasers was studied by correlating the calculated laser-irradiated area on the cylindrical material’s surface to the experimentally determined material removal rate. The mathematical analysis included the calculation of the coordinates of each individual pulse, the respective spatial dimensions of each pulse on the curved surface and, thus, the resulting pulse-to-pulse overlap and a mapping of the accumulated intensity distribution. Although the demonstrated experiments only represent a fraction of the possible parameter combinations, they provide a solid impression of the fundamental processing characteristics of laser turning with ultrashort laser pulses.

## 2. Materials and Methods

In the practical experiments, a laser system with a pulse duration of 900 fs, a wavelength of 1030 nm and circular polarization was applied (TruMicro 5050 femto edition, Trumpf SE & Co. KG, Ditzingen, Germany). Three linear axes were used for positioning the focused laser beam on the specimen, and one rotary axis was deployed for rotating the work piece at a constant speed. The processing head was equipped with a focusing lens with a 60 mm focal length, generating a focal spot diameter of 25 µm. As a test material, Stellite rods made of 88% tungsten carbide and with a flexural strength of 4000 N/mm^2^, with radii of 0.5, 0.7 and 1 mm, were used. The practical results for calculating the ablation rate and measuring the laser spot dimensions on the material’s surface were evaluated using a confocal laser scanning microscope (VK-X3000, Keyence, Osaka, Japan). To calculate the material’s ablation rate, the resulting cross-sectional area of the cylindrical material was subtracted from the initial area and, subsequently, multiplied by the linear feed rate of the laser movement. Please note that in this contribution, the investigated geometrical conditions of the process resulted in a maximum angular laser impingement of 72°.

The first set of experiments consisted of punctual ablation tests on a steady Stellite rod. Here, the lateral positioning of the focused laser beam gradually changed. The subjects of the evaluation were the dimensions of the laser imprint on the cylindrical material’s surface in order to determine the accuracy of the calculated behavior. The second set of experiments was conducted under a set of constant conditions, such as a rotation speed of 500 min^−1^, pulse frequency of 50 kHz, pulse energy of 350 µJ and a linear laser feed rate of 0.07 mm/s. As an altering parameter, the lateral positioning of the laser beam, as well as the positioning of the focal plane on the material, gradually changed for the three different work piece diameters. The reduction in the material’s diameter was interpreted as the material removal rate, corresponding to the ablated material volume and the linear feed rate of the laser. The third set of experiments consisted of laser turning experiments while using a rotating trepanning optic. Here, two different feed rates and the resulting beam path on the specimen were compared, and the effect on the surface formation is discussed.

**Figure 1:**
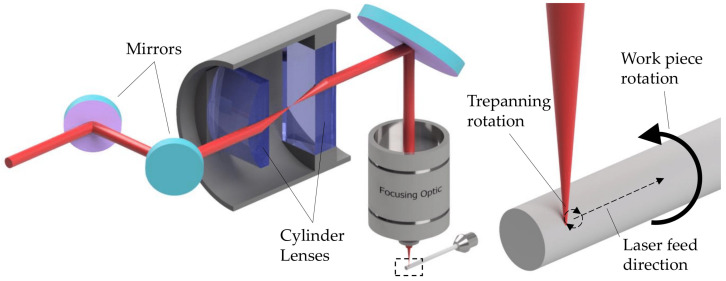
Illustration of the tangential laser turning process using a trepanning optic with two crosswise arranged cylinder lenses.

Figure 1 shows the conceptual set-up of the used trepanning optic according to [31]. It consisted of two cylinder lenses that were arranged at a 90° angle to each other at a fixed, confocal distance. The simultaneous rotation of these lenses realized the trepanning motion (rotation speed of 14,000 rpm, leading to a focal point revolution speed of 28,000 rpm), while the trepanning diameter and pitch angle could be controlled by the two motorized mirrors in front of the cylinder’s lens bundle.

The basis of the considerations in this contribution are the geometric conditions of the process set-up and the focused laser beam propagation. The influence of the work piece dimensions, the beam propagation, the laser positioning and the consequent effects on the laser turning process can be described as follows.

**Figure 2:**
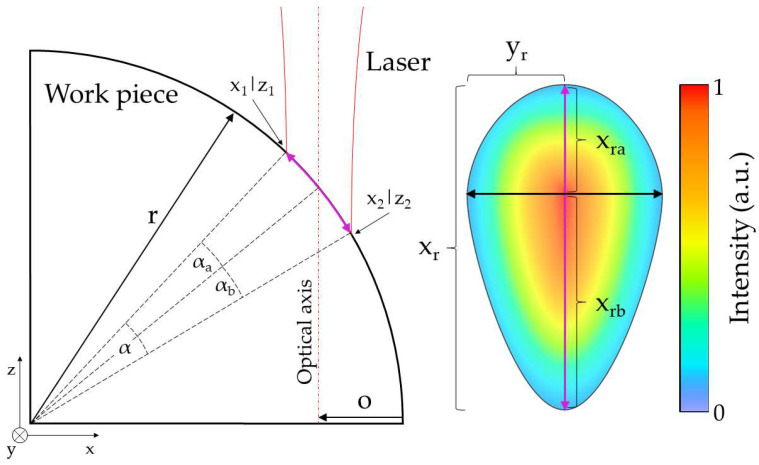
Illustration of a focused laser beam on a cylindrical surface and the resulting elliptical intensity distribution.

If the focal plane of the laser is considered to be stable at the rotational plane of the work piece, altering the positioning of the optical axis of the laser beam on the cylindrical specimen causes a distortion of the irradiated area, as described in [17]. The resulting laser spot on the surface appears as an ellipse-shaped area with its axes intersecting each other off center, as shown in Figure 2. Variables in figure are explained in Nomenclature.

The laser-irradiated area on the material’s surface is calculated by adding the areas of the upper and lower semi-ellipse:

(1) $$A=\frac{\left(x_{ra}\cdot y_{r}\cdot \pi \right)}{2}+\frac{\left(x_{rb}\cdot y_{r}\cdot \pi \right)}{2}=\frac{x_{r}}{2}\cdot y_{r}\cdot \pi$$

Please note that the calculation of the area of the nonsymmetric ellipse, as shown in Figure 2, offers the same result as calculating the area of a symmetrical ellipse with $x_{ra}=x_{rb}$. To calculate the length of the long semi-axes $x_{ra}$ and $x_{rb}$, the points of intersection of the focused laser beam and the cylindrical material within a Cartesian coordinate system with its origin on the rotation center point need to be determined. This can be conducted by subtracting the circle function from the function of the laser beam edges and solving the equations for z with $z_{r}$ as the Rayleigh length and $f_{s}$ as a potential shift of the focal plane:

(2) $$\left(r-o\right)-w_{f}\cdot \sqrt{1+{\left(\frac{z_{1}-f_{s}}{z_{R}}\right)}^{2}}-\sqrt{r^{2}-z^{2}}=0,\;\;\;\;\;for\;0<z_{1}<r\;and\;w_{f}<o<r-w_{f}$$

(3) $$\left(r-o\right)+w_{f}\cdot \sqrt{1+{\left(\frac{z_{2}-f_{s}}{z_{R}}\right)}^{2}}-\sqrt{r^{2}-z^{2}}=0,\;\;\;\;\;for\;0<z_{2}<r\;and\;w_{f}<o<r-w_{f}$$

The offset value, o, describes the distance of the optical axis of the laser beam to the material’s lateral surface. The corresponding $x_{1/2}$ values are calculated, subsequently, according to Pythagorean’s theorem by:

(4) $$x_{1/2}=\sqrt{r^{2}-{z_{1/2}}^{2}}$$

The distance of the points of intersection of the focused laser beam on the cylindrical material can now be determined by applying a cylindrical surface unwrapping using the two *x*-coordinates, the radius, r, and the angle, α, that describes the angular difference between the two points with respect to the origin:

(5) $$x_{r}=x_{ra}+x_{rb}=\frac{\left(\frac{\alpha_{a}}{180}\right)\cdot \pi \cdot r}{2}+\frac{\left(\frac{\alpha_{b}}{180}\right)\cdot \pi \cdot r}{2}=\frac{\left(\frac{\alpha }{180}\right)\cdot \pi \cdot r}{2}$$

(6) $$\alpha =\alpha_{a}+\alpha_{b}=\left(\cos^{-1}\left(\frac{x_{1}}{r}\right)-\cos^{-1}\left(\frac{r-o}{r}\right)\right)+\left(\cos^{-1}\left(\frac{r-o}{r}\right)-\cos^{-1}\left(\frac{x_{2}}{r}\right)\right)=\left(\cos^{-1}\left(\frac{x_{1}}{r}\right)-\cos^{-1}\left(\frac{x_{2}}{r}\right)\right)$$

The length of the short half-axis is considered to be the beam radius at the respective *z*-value, where the optical axis intersects with the material’s surface, whereby $w_{f}$ is the focal spot radius [32]:

(7) $$y_{r}=w_{f}\cdot \sqrt{1+{\left(\frac{z}{z_{r}}\right)}^{2}}$$

With the calculated area and dimensions of the half-axes, the resulting pulse overlap depends on the pulse repetition rate, f, the rotation speed, u, and linear feed rate, v. It can be determined as follows:

(8) $${OL}_{x}=1-\left(\frac{r\cdot \pi \cdot u\cdot \frac{1}{f}}{2\cdot \frac{x_{r}}{2}}\right)=1-\left(\frac{r\cdot \pi \cdot u\cdot \frac{1}{f}}{x_{r}}\right)$$

(9) $${OL}_{y}=1-(\frac{\frac{v}{u}}{2\cdot y_{r}})$$

Using $\frac{x_{r}}{2}$ as the long half-axis of the ellipse shape, in Equation (8) it is considered an adequate approximation, since the calculation of the area of the symmetrical and nonsymmetrical ellipse results in the same outcome.

Equations (1)–(9) describe a laser turning process consisting of two homogeneous movements, as well as the rotation speed of the work piece and the linear feed rate of the laser in *y*-direction. When applying a rotating trepanning optic, however, several additional considerations have to be added to the calculations. The superimposed motion of the circular beam movement, the work piece rotation and the laser feed may result in a beam path pattern that affects the surface of the laser machined product. Not only does the irradiated area of the laser beam change according to the applied trepanning radius, as described in [17], but the pulse-to-pulse distance on the material’s surface also depends on the trepanning rotation speed and direction, potentially causing a patterned excessive energy input onto the material. The coordinates of the laser pulses on the material’s surface within one trepanning rotation can be calculated as follows:

(10) $$x=\cos\left(\cos^{-1}\left(\frac{r-o-\sin\left(\varphi_{t}\right)\cdot t_{r}}{r}\pm \frac{u}{f}\right)\right)\cdot r$$

(11) $$z=\sin\left(\cos^{-1}\left(\frac{r-o-\sin\left(\varphi_{t}\right)\cdot t_{r}}{r}\pm \frac{u}{f}\right)\right)\cdot r$$

(12) $$y=\frac{v}{f}+\cos\left(\varphi_{t}\right)\cdot t_{r}$$

Note that the ± operator in Formulas (10) and (11) depends on the rotation direction of the work piece. Also, the cosine function in (12) represents a clockwise trepanning motion. For a counterclockwise rotation, a sinus function needs to be applied.

## 3. Results

**Figure 3:**
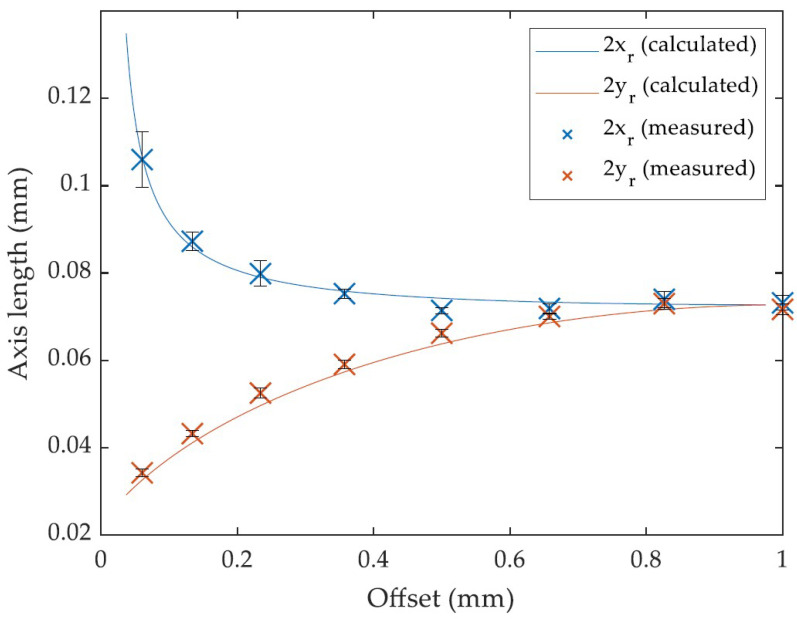
Calculated and measured laser imprints on a cylindrical work piece. Radius: 1 mm, pulse energy: 50 µJ, repetition rate: 0.25 kHz, number of pulses: 1000 and number of imprints: *n* = 5.

To validate the calculated dimensions of the projected laser spot area on the surface of the cylindrical material, a study on a steady work piece with a radius of 1 mm over an increasing offset was conducted. For each examined offset value, the extent of the laser imprint on the material’s surface in the *x*-direction (i.e., offset/rotation direction) and *y*-direction (i.e., laser feed direction) was measured under a laser scanning microscope. Each measurement point is represented as the average of five measurements on five separate laser imprints. One laser imprint was generated by applying 1000 pulses with a pulse energy of 50 µJ and a repetition rate of 0.25 kHz. The results are shown in Figure 3.

The calculations, as well as the measurements, show a large dilation of the $x_{r}$-axis towards the edge of the material (i.e., small offset values), whereas the $y_{r}$-axis displays a contraction towards a nominal, in-plane focus diameter of 25 µm. For the larger offset values, the two curves constantly approach each other to the point of a maximum offset, in this case 1 mm, where the resulting $x_{r}$- and $y_{r}$-axes were equal in length, which represents a circular laser imprint at a perpendicular impingement (cf. 0°) on the material’s surface, with a corresponding defocusing. As the measurement values and the calculated values show an overall good conformity, the assumed calculations can be treated as suitable henceforth.

As described in (1), the laser-irradiated area on the material and, therefore, the reachable laser fluence, depend on the dimensions of the $x_{r}$- and $y_{r}$-axes. This means that at an offset value of 0.05 mm, the laser-irradiated area calculates to approximately 2.98 × 10^−5^ cm^2^ compared to 4.91 × 10^−6^ cm^2^ in the actual focal plane, i.e., more than five times as large and, hence, less than one-fifth of the laser fluence. In addition, the reflectance of the material at the applied laser wavelength needs to be taken into account. According to the findings of Lickschat et al. [8], the reflectance of cemented tungsten carbide amounts to approximately 0.46 for incident angles lower than 78° at an applied wavelength of 1030 nm, with a circular polarization (as applied here). This is considered a comparative value, because the used material in this study consisted of 88% tungsten carbide. As already mentioned in Section 2, the highest incident angle for the studies in this contribution was found for a material radius of 1 mm and an offset value of 0.05 mm, resulting in a maximum incident angle of approximately 72°. Hence, the calculated fluence on the material’s surface has to be multiplied with a factor of 0.54 to mimic the actually absorbed share of the laser power.

### 3.1. Laser Turning with a Steady Beam

**Figure 4:**
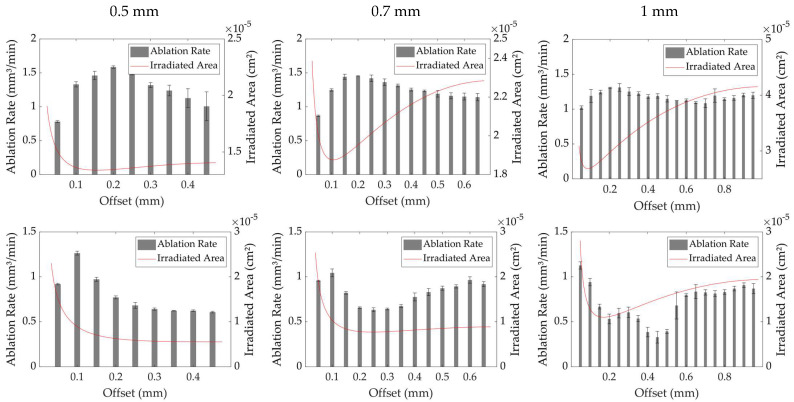
Overlay of the laser-irradiated area and the ablation rate over an increasing offset for three different work piece radii: (**Top**) focal plane on work piece rotation center level; (**Bottom**) focal plane shift 0.37 mm upward.

To illustrate the effect of the laser beam positioning on the cylindrical material in the laser turning process, a series of studies of the ablation rate at increasing offsets for different work piece diameters was conducted. Three distinct work piece radii of 0.5, 0.7 and 1 mm were prepared in advance and, subsequently, machined with the focal plane matching the level of the rotation center of the work piece. The results are summarized in Figure 4 (Top). The calculated area that was irradiated by the laser for the increasing offset is indicated by a red line. The graphs show that the highest ablation rate for all three reviewed diameters was found at an offset value of approximately 0.2 mm. The overall value of the peak ablation rates, however, appeared to decrease with a growing material diameter from 1.59 mm^3^/min to 1.26 mm³/min. The incident angle at an offset value of 0.2 mm for a work piece radius of 0.5 mm calculated to approximately 36.9°. Projecting this 36.9° angle onto the other work piece diameters resulted in offset values of 0.28 mm for a radius of 0.7 mm and 0.4 mm for a radius of 1 mm, respectively. Comparing the graphs at these offset values also showed a decrease in the ablation rate for larger work piece radii. This behavior is linked to the increasing angle of incidence and the resulting increase in reflectance of the laser radiation off the material’s surface, as well as the level of defocusing and the associated loss in fluence. The low point of the calculated laser area on the surface on the other hand showed a continuous shift towards smaller offset values for an increasing material diameter. The overall low point value of the area increased from 1.33 × 10^−5^ cm^2^ for a 0.5 mm radius to 2.64 × 10^−5^ cm^2^ for a 1 mm radius. This doubling of the effective area decreased the laser fluence on the material and, hence, reduced the achievable ablation rate. Apparently, the offset values of the lowest irradiated area for each material diameter did not match the offset values of the highest ablation rates. This suggests that in the tangential processing of cylindrical materials, a larger area in conjunction with an angular incidence is beneficial to the process speed. The results shown in Figure 4 (Top) suggest that a shift of the focal plane to the corresponding *z*-level of the low point of the irradiated area may increases the ablation rate due to the higher reachable fluences. Hence, a study with similar parameters but with a shift of the laser focal plane 0.37 mm upwards, corresponding to the Rayleigh length, was conducted. The results are provided in Figure 4 (Bottom). The calculations of the irradiated area reveal that the values of the laser-irradiated area’s low points dropped substantially, which, in turn, increased the laser fluence on the surface. For a material radius of 0.5 mm, a distinct low point could not be achieved; however, at a maximum offset, the irradiated area was less than half as large as without the focal displacement. A similar decrease in the irradiated area at the respective distinct low points could be observed for the other inspected material radii. For work piece radii of 0.5, 0.7 and 1 mm, the offset values of the distinct low points without a shift of the focal plane were 0.156, 0.106 and 0.083 mm, respectively. Adding a shift of 0.37 mm to the focal plane changed these offset values to 0.5, 0.246 and 0.170 mm, respectively. The offset values of the area low points appeared to shift towards the material’s center, suggesting a higher fluence and, therefore, higher ablation rates at larger offsets. The ablation rate peaks, however, apparently shifted in the opposite direction towards the material edge, contradicting this assumption. It does, however, coincide with the findings of Chang et al. [33], who stated that a defocused laser machining process with ultrashort pulses on the material’s surface leads to a higher material removal rate. The study explains that the highest material removal rate is found at $\left(z_{top}^{2}+z_{r}^{2}\right)/\left(z^{2}+z_{r}^{2}\right)=e^{2}$, with z as the focal plane shift, $z_{top}$ as the distance between the peak point of the laser-affected volume and the focal plane and $z_{r}$ as the Rayleigh length. The laser-affected volume in this case is the entire volume of the focused laser beam, where the energy density is above the ablation threshold fluence. Defocusing leads to a larger irradiated area on the material’s surface, thus lower fluences, as well as higher pulse-to-pulse overlaps. According to Lickschat et al. [8], the removal efficiency for cemented tungsten carbide peaks at 2.7 J/cm^2^ and decreases for higher values. With applied fluences of 26.0 J/cm^2^ for a work piece of a 0.5 mm radius at an offset value of 0.2 mm and 17.9 J/cm^2^ for a work piece of 0.7 mm and 11.6 J/cm^2^ for a work piece of 1 mm radius, the fluence shifts towards a more efficient regime.

**Figure 5:**
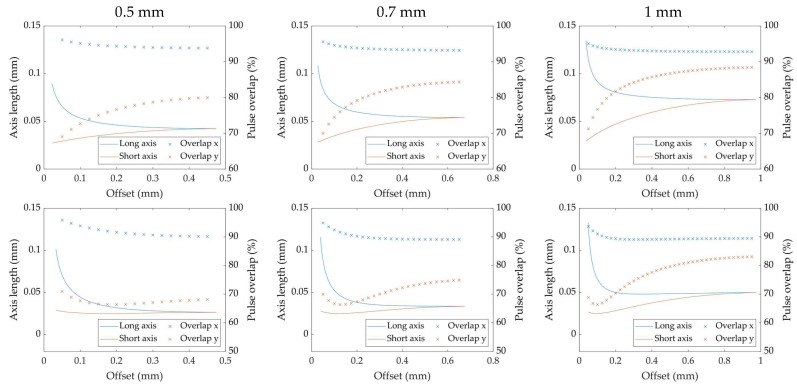
Overlay of the axes dimensions and pulse-to-pulse overlaps over an increasing offset for three different work piece radii: (**Top**) without focal plane shift; (**Bottom**) with focal plane shift of 0.37 mm.

In addition, the overall ablation rate revealed a decrease for all examined material radii, when a focal shift of 0.37 mm was applied. This effect can be ascribed to the behavior of the long and short axes of the ellipsoid, which represents the laser-irradiated area for each individual laser pulse. According to the work piece radius and the applied offset value in the process, the dimensions of the axes change significantly. Figure 5 provides the calculated length of each axis for the examined work piece radii with and without a focal plane shift of 0.37 mm. Additionally, the resulting pulse-to-pulse overlap in the *x*-direction (i.e., rotation direction) and *y*-direction (i.e., laser feed direction) is marked. With the general conditions of the experiments, such as work piece rotation speed and diameter, pulse repetition rate and linear feed rate, held constant, the pulse-to-pulse overlap values solely depend on the positioning of the optical axis of the laser beam on the material. The overlap value in the y-direction is mainly affected by the extent of the minor axis, $y_{r}$, as described in (7). An increasing offset value and, therefore, an increase in the defocusing, leads to a larger $y_{r}$-axis, causing larger overlap values. The overlap value in the x-direction, on the other hand, greatly depends on the extent of the $x_{r}$-axis, which stands in correlation with the material diameter, hence, the curvature of the surface. Small offset values lead to large $x_{r}$ values, resulting in large overlap ratios. The graphs for the three inspected material radii with and without a shift of the focal plane, as shown in Figure 5, indicate that the overlap values in the *x*-direction for an increasing material radius are hardly affected by the extent of the $x_{r}$-axis, despite the rather large change in length. This is due to the increasing surface speed for a larger material radius. The overlap in the y-direction, however, appears to be rather strongly affected by the corresponding $y_{r}$-dimension. A distinct increase in the pulse-to-pulse overlap can be observed for the increasing offset values and $y_{r}$ length, respectively, for the focal plane matching the rotation center. The shift in the focal plane results in a downward spike of the overlap values for the x-direction, which grows more pronounced for larger material radii. This downward spike in the pulse-to-pulse overlap in the y-direction is directly caused by the focused beam propagation of the laser. The upwards shift of the focal plane causes the beam waist to strike the material’s surface at a higher offset value, creating a smaller irradiated area with a higher fluence but also decreasing the overlap value for this spatial direction. This behavior along with the overall decrease in the pulse overlap for both spatial directions appears to be the reason for the reduced material ablation rate when shifting the focal plane above the material’s rotational center.

### 3.2. Laser Turning Using a Trepanning Optic

**Figure 6:**
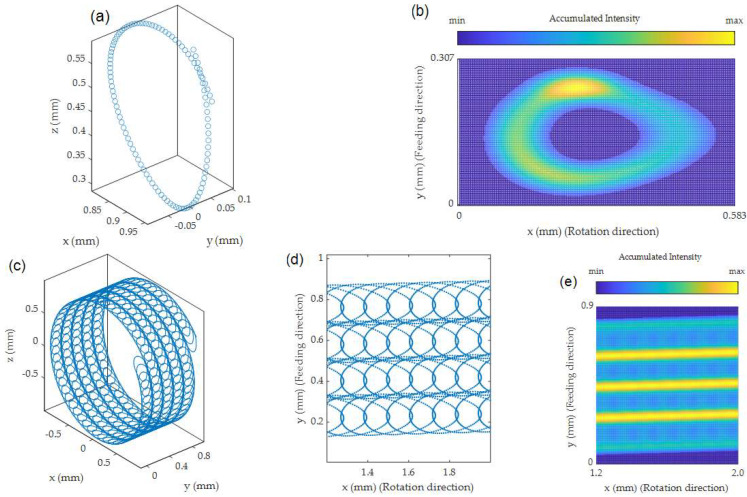
(**a**) Pulse coordinates within a single trepanning circulation; (**b**) accumulated laser intensity of each individual pulse within a trepanning circulation after applying a cylindrical surface unwrapping; (**c**) pulse coordinates for five consecutive work piece rotations; (**d**) cylindrical surface unwrapping of selected pulse coordinates onto a plane’s surface; (**e**) accumulated laser intensity pattern of several work piece rotations. Parameters: *r* = 1 mm, *v_u_* = −500 min^−1^, *v* = 1.5 mm/s, *t_r_* = 0.1 mm, *t_v_* = 28,000 min^−1^, *o* = 0.15 mm and *f* = 50 kHz.

Using a trepanning optic is very common in laser drilling operations. Because of the fast beam deflection, the flexible adjustment of the trepanning diameter and the impassivity to noncircular beam shapes, it is a popular tool to rapidly realize boreholes. Some of these advantages can also be employed in the laser turning process. As shown in [17], the use of a trepanning optic in laser turning can significantly improve the material ablation rate and, therefore, the processing time. The main aspect herein is the spatial separation of the individual laser pulses, which prevents particle shielding to a certain degree. This processing approach, however, may result in inadvertent surface structures that may compromise the functionality of the work piece. In order to predetermine the resulting laser beam path and, therefore, the pattern on the material’s surface, Equations (10)–(12) in combination with (5) and (7) can be used to adjust the process parameters. A case example of such a laser beam pattern on a cylindrical surface in the laser turning process using a trepanning optic is shown in Figure 6. To obtain an impression of the accumulated laser intensity distribution on the material throughout the process, the calculated pulse coordinates are augmented by a Gaussian intensity profile for each individual laser spot in compliance with the calculated dimensions from (5) and (7).

The illustration in Figure 6a shows the coordinates of the intersection point of the optical axis of the laser beam with the spinning material’s surface within one trepanning rotation, which in this example are 107 pulses (rounded). The start and end points of the trepanning motion are approximately *y* = 0.05 mm, correlating to the trepanning radius. The end point has a slightly larger *y*-value due to the feeding motion of the laser beam. The visible overreach of the start and end points in *x*-direction is caused by the rotation speed of the material. This rotation speed, together with the trepanning rotation speed, also causes a difference in the pulse-to-pulse distance throughout one trepanning motion, meaning there was a different energy input into the material at all irradiated points. Additionally, the ratio of the rotation movement of the work piece and the trepanning speed caused the projected loop on the surface to distort. Slower trepanning speeds will make it appear smaller, whereas, at the same time, generating fewer loops within one work piece rotation.

When each individual laser pulse coordinate of this single trepanning motion is augmented with a Gaussian laser beam profile and their intensities are added up, an intensity map, as shown in Figure 6b, is the result. Please note that the coordinates were transitioned to a plane by applying a cylindrical surface unwrapping beforehand. The y-dimension of the resulting loop was roughly 250 µm, which is slightly larger than the applied trepanning diameter of 200 µm. This enlargement was caused by the defocusing of the laser due to the curved surface of the material in combination with the chosen offset value (cf. Figure 2). The *x*-dimension of the irradiated area stretched out to roughly 500 µm, which is much larger than the trepanning diameter. This behavior was mainly caused by the proximity to the material’s edge (e.g., low offset values), which resulted in a strong distortion of the $x_{r}$-axis of the projected laser beam on the cylindrical surface (cf. Figure 3). This area also showed the lowest laser intensity input throughout the trepanning motion, since the rather large, irradiated area resulted in a low laser fluence. The highest intensity accumulation in this case was found in the area where the loop of the trepanning motion closed because of multiple laser exposures. The second highest intensity accumulation was located at the bottom left curve of the projected loop. In that area the trepanning motion and the rotation motion of the work piece were in the same direction, causing a higher pulse-to-pulse overlap, thus increasing the accumulated intensity input.

Extending this single trepanning rotation for multiple work piece rotations provides an impression of the actual laser beam path on the material’s surface in the laser turning process, as shown in Figure 6c. Considering the work piece rotation speed and the linear feeding rate of the laser beam, an extensive prediction of the processing result can be made. The cylindrical surface unwrapping and the subsequent augmentation deliver a precise outline of which areas of the material’s surface experience the most intense laser exposure (cf. Figure 6d,e).

**Figure 7:**
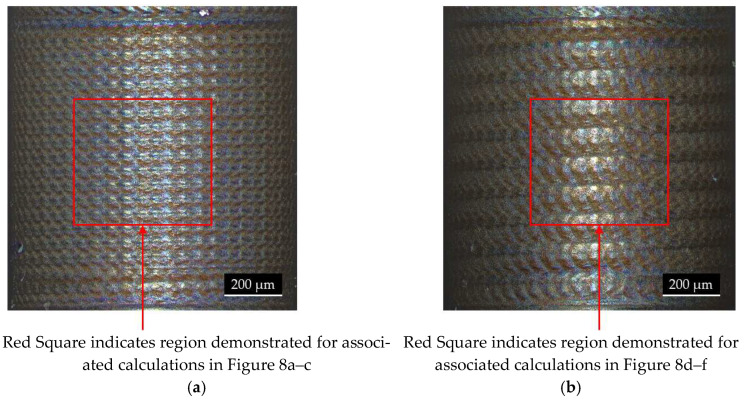
Microscope images of the resulting surface patterns after the laser turning process with a trepanning optic: (**a**) feed rate of 0.417 mm/s; (**b**) feed rate of 0.834 mm/s. *r*: 0.5 mm, *u*: 500 rpm, *t_r_*: 0.05 mm, *t_v_:* 28,000 rpm, *o*: 0.1 mm, *f*: 50 kHz and pulse energy: 200 µJ.

**Figure 8:**
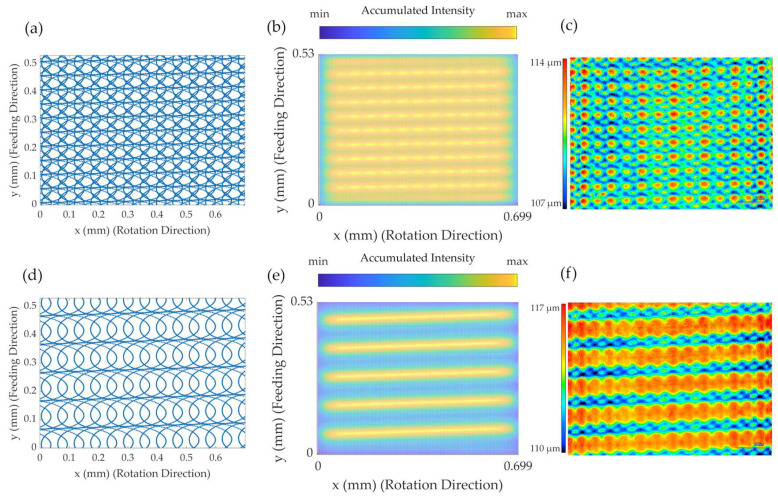
Illustration of the calculated beam path, calculated accumulated laser intensity and laser scanning microscope image (height) of the laser turning process with a trepanning optic: (**a**–**c**) feed rate of 0.417 mm/s; (**d**–**f**) feed rate of 0.834 mm/s. *r*: 0.5 mm, *u*: 500 rpm, *t_r_*: 0.05 mm, *t_v_*: 28,000 rpm, *o*: 0.1 mm, *f*: 50 kHz and pulse energy: 200 µJ.

The actual laser machined surface and the associated calculations are illustrated in Figure 7 and Figure 8. They show the calculation of the laser beam path and the consequential accumulation of the laser intensity on the material, as well as the actual laser machined surface for the distinct set of parameters for two different feed rates.

The beam path for the slower feed rate of 0.417 mm/s appeared as a relatively homogeneous pattern, leaving white spaces where the optical axes of the laser beam did not pass over the surface. The corresponding intensity accumulation map, however, showed that these spots were irradiated by the laser as well, but with relatively low intensity values. Therefore, these spots were rather “carved out” of the material, which left them as a material cluster on the remaining surface. This pattern can be seen on the actual height profile of the laser machined material, as seen in the depiction Figure 8c. By altering the feed rate, the overlap ratio of the trepanning loops over one work piece rotation was varied. If the trepanning radius and work piece rotation speed in the process happen to coincide with a certain feed rate in such a way that the edges of the trepanning motion overlie with the edge of one of the previous work piece rotations, the dotted surface pattern merges into a line. The accumulated intensity map strongly visually emphasizes this condition. The areas where the trepanning loop edges overlap each other experience the most laser exposure time, as well as the highest laser intensity dose, creating a distinct groove in the material’s surface. The beam path outside of these explicit lines still experience laser intensity, only at a much smaller accumulated dose, but this can also be seen in the intensity map. Again, the white areas in the laser beam path diagram are areas that are carved out, visible as small material remains inside of the groove. The angle and distance of the formed groove are determined by the trepanning radius, work piece rotation speed and feed rate under the premise that the edges of the trepanning loops match up after one work piece rotation.

## 4. Discussion

The consideration of the laser-irradiated area and, therefore, the laser fluence on a cylindrical surface in the laser turning process and the resulting ablation behavior is a complex matter. The geometrical conditions, such as work piece radius and beam propagation, as well as dynamic aspects, including rotation speed, beam movement and pulse repetition rate, have to be taken into account. The presented results make apparent that the resulting laser fluences on the surface of a cylindrical work piece in the laser turning process are far lower than when processing on an even surface in the focal plane. An adjustment of the focal plane may increase the reachable fluence on the curved surface; however, the resulting drop in irradiated area and the consequentially lower pulse overlap appear to be deleterious to the material removal rate. Furthermore, the offset value corresponding to the smallest reachable irradiated area appears to differ from the offset value that generates the highest ablation rate. This also suggests that in the laser turning process a larger irradiated area at a constant pulse energy is more beneficial to the reachable ablation rate. Studies by Chaja et al. [34] have shown that for an increasing spot size, the maximum specific removal rate for a Gaussian beam shape decreases almost linearly. This observation, however, was made for applying multiple laser pulses to the same point. Bischoff et al. [35], on the contrary, state that for larger spot radii, an increase in the material removal rate can be accomplished using faster possible scanning speeds for constant pulse overlap values.

It is notable that the offset value for the highest achievable ablation rate for the process with no shift in the focal plane was larger than the offset value for the irradiated area’s low point, and during the process with an adjusted focal plane level, the offset value of the highest ablation rates was lower than the offset values of the corresponding area’s low point. This change in orientation of the maximum ablation rate towards the area’s low points was most likely the result of the focal plane shift.

As seen from all of the conducted experiments, a larger irradiated area appeared to be beneficial for the ablation rate in the process. With the applied focal shift of 0.37 mm, the largest irradiated area was to be found at lower offset values, rather than higher ones and, therefore, the maximum ablation rate was found at these values. This, however, only applied to a certain degree until the impingement angle was too low and the reflection of the surface countered this effect. To further enhance the accuracy of the intensity distribution model that is presented in this contribution, the consideration of the absorption and reflection behavior of the used material in dependence of the impingement angle would prove beneficial.

While the use of a trepanning optic is certainly capable of increasing the processing speed, it comes, however, with the condition that the work piece surface experiences a structuring due to excessive patterned laser exposure to some degree. The resulting pattern depends on a large variety of parameters, such as the trepanning speed and radius, the work piece radius and rotation speed, the feeding speed and the applied offset value. Additionally, a variety of different fluences applies to the material’s surface within every trepanning rotation according to the geometrical conditions. Furthermore, it has to be taken into account that after each processing step, the work piece diameter is reduced and the resulting beam path on the surface changes entirely. These circumstances make it elaborate to predict the entire process of a multistep laser turning procedure. However, knowing the underlying geometrical proceedings and ablation behaviors that are presented in this contribution can help avoid any undesired processing results and may improve the overall outcome in the tangential laser turning approach.

## 5. Conclusions

In this contribution, the laser beam path on a cylindrical material’s surface and, hence, the laser energy distribution during the tangential laser turning process using ultrashort laser pulses was analyzed. The geometrical conditions of the process set-up, as well as the dynamic aspects of this laser micromachining approach, against the background of the focal plane positioning and the use of fast beam deflection using a trepanning optic were discussed. By calculating the irradiated area of the focused laser beam on the material’s surface and subsequent augmentation with a Gaussian intensity distribution, a model for an energy distribution map was presented. In summary, the key findings are:

- The distortion of the laser-irradiated area on a cylindrical surface had a large impact on the available fluence on the material, as well as on the pulse-to-pulse overlap and, therefore, on the ablation rate;
- The highest ablation rate for the laser turning with a steady beam was found at an offset value that differed from the point with the lowest irradiated area and accordingly highest fluence;
- A shift of the focal plane away from the work piece rotation center was deleterious to the material removal rate;
- Using a trepanning optic in the laser turning process resulted in a patterned surface of the material;
- The trepanning parameters need to be carefully adjusted to the geometrical conditions of the process in order to minimize the significance of the surface pattern.

Further studies may include an evaluation of the absorption and reflection of the laser energy under various impingement angles and resulting effects on the energy distribution in the tangential laser turning process.

## Data Availability

The data underlying the results presented in this paper are not publicly available at this time but may be obtained from the authors upon reasonable request.

## Nomenclature

*r*	Work piece radius
*o*	Offset (distance of the optical axis/trepanning center to work piece)
*x_r_*	Long half-axis of ellipse
*y_r_*	Short half-axis of ellipse
*A*	Area of the laser irradiation on the material’s surface
*w_f_*	Focal spot radius
*z_r_*	Rayleigh length
*x, y, z*	Cartesian coordinates of the laser pulses on the material’s surface
*OL_x/y_*	Laser pulse overlap
*v*	Linear feed rate of the laser beam
*u*	Rotation speed of the work piece
*t_r_*	Trepanning radius
*t_v_*	Trepanning speed
*f*	Laser repetition frequency
*ϕ* *_t_*	Angular difference between two consecutive laser pulses within a trepanning circulation
*f_s_*	Focal plane shift (>0)
